# Evaluation of LDBio *Aspergillus* ICT Lateral Flow Assay for IgG and IgM Antibody Detection in Chronic Pulmonary Aspergillosis

**DOI:** 10.1128/JCM.00538-19

**Published:** 2019-08-26

**Authors:** Elizabeth Stucky Hunter, Malcolm D. Richardson, David W. Denning

**Affiliations:** a Division of Infection, Immunity and Respiratory Medicine, Faculty of Biology, Medicine and Health, Manchester Academic Health Science Centre, University of Manchester, Manchester, United Kingdom; b National Aspergillosis Centre, Manchester University NHS Foundation Trust, Manchester, United Kingdom; c Mycology Reference Centre Manchester, Manchester University NHS Foundation Trust, Manchester, United Kingdom; Carter BloodCare & Baylor University Medical Center

**Keywords:** *Aspergillus* serology, aspergillosis, chronic pulmonary aspergillosis, lateral flow assay

## Abstract

Detecting *Aspergillus*-specific IgG is critical to diagnosing chronic pulmonary aspergillosis (CPA). Existing assays are often cost- and resource-intensive and not compatible with resource-constrained laboratory settings. LDBio Diagnostics has recently commercialized a lateral flow assay based on immunochromatographic technology (ICT) that detects *Aspergillus* antibodies (IgG and IgM) in less than 30 min, requiring minimal laboratory equipment. A total of 154 CPA patient sera collected at the National Aspergillosis Centre (Manchester, United Kingdom) and control patient sera from the Peninsula Research Bank (Exeter, United Kingdom) were evaluated. Samples were applied to the LDBio *Aspergillus* ICT lateral flow assay, and results were read both visually and digitally. Results were compared with *Aspergillus* IgG titers in CPA patients, measured by ImmunoCAP-specific IgG assays. For proven CPA patients versus controls, sensitivity and specificity for the LDBio *Aspergillus* ICT were 91.6% and 98.0%, respectively. In contrast, the routinely used ImmunoCAP assay exhibited 80.5% sensitivity for the same cohort (cutoff value, 40 mg of antigen-specific antibodies [mgA]/liter). The assay is easy to perform but challenging to read when only a very faint band is present (5/154 samples tested). The ImmunoCAP *Aspergillus* IgG titer was also compared with the *Aspergillus* ICT test line intensity or rate of development, with weak to moderate correlations. The *Aspergillus* ICT lateral flow assay exhibits excellent sensitivity for serological diagnosis of CPA. Quantifying IgG from test line intensity measurements is not reliable. Given the short run time, simplicity, and limited resources needed, the LDBio *Aspergillus* ICT is a suitable diagnostic tool for CPA in resource-constrained settings.

## Abstract (precis)

Detecting *Aspergillus*-specific IgG is critical to diagnosing chronic pulmonary aspergillosis (CPA). Existing assays are often cost- and resource-intensive and not compatible with resource-constrained laboratory settings. LDBio Diagnostics has recently commercialized a lateral flow assay based on immunochromatographic technology (ICT) that detects *Aspergillus* antibodies (IgG and IgM) in less than 30 min, requiring minimal laboratory equipment.

## INTRODUCTION

Chronic pulmonary aspergillosis (CPA) is usually a progressive fungal disease, most often complicating other respiratory disorders. The majority of cases are secondary to pulmonary tuberculosis (TB) and chronic obstructive pulmonary disease. There are an estimated 3 million CPA cases worldwide (1). CPA is associated with severe morbidity and mortality (2), but outcomes can be improved with long-term antifungal therapy or surgery (3). Accurate diagnosis of CPA can be difficult, however, due to heterogeneity of symptoms and similarity to other chronic respiratory conditions, notably, mycobacterial infection (4), and also due to the fact that no single diagnostic test is sufficient for a clear diagnosis of CPA. Rather, diagnosis relies on a combination of clinical symptoms, radiological findings, and microbiological evidence (5).

Serology is perhaps the most important and reliable component of the CPA diagnostic pathway (5–9). One of the most common methods for detecting *Aspergillus*-specific antibodies in patient sera is the precipitins assay (10, 11), typically conducted by the use of Ouchterlony agar gel double diffusion or counterimmunoelectrophoresis. Though widely considered a standard assay, the precipitins method has disadvantages, including a long turnaround time and poor interlaboratory reproducibility and standardization (12, 13). Other serological assays are commercially available, such as indirect hemagglutination and enzyme-linked immunosorbent assay (ELISA)/enzyme immunoassay (EIA) (14), but levels of performance differ between tests, and redefinition of cutoff values for distinct populations and diagnoses may be necessary to optimize performance (6). Furthermore, these assays are often costly and require sophisticated equipment, making them unsuitable for use in low- and middle-income countries where tuberculosis prevalence is high (15) and CPA diagnostics are a critical necessity for early recognition of CPA complicating TB and for distinguishing between these similarly presenting conditions.

LDBio Diagnostics (Lyons, France) has introduced a new point-of-care lateral flow assay (LFA) (LDBio *Aspergillus* ICT) for detection of *Aspergillus* antibodies (IgG and IgM). The assay utilizes immunochromatographic technology (ICT) and has recently been validated against a spectrum of *Aspergillus*-related diseases (16), including a moderate number (*n* = 79) of CPA cases. It has been demonstrated to meet the ASSURED (“affordable, sensitive, specific, user-friendly, rapid and robust, equipment-free, and deliverable to end users”) criteria outlined by the World Health Organization (16) and therefore would be compatible with resource-constrained settings. In this study, we evaluated the performance of the LDBio *Aspergillus* ICT LFA as a rapid serological test specifically for CPA diagnosis, using serum from patients with known CPA and case-matched healthy controls. We compared this assay to our routine serological test (ImmunoCAP *Aspergillus*-specific IgG EIA), which is used alongside other CPA diagnostic criteria, as well as to the LDBio *Aspergillus* Western blot (WB) (13) immunoblot assay and determined the potential for quantitative interpretation of the ICT test result.

## MATERIALS AND METHODS

### Serological samples.

This study was performed by convenience sampling from 154 CPA patients identified at the National Aspergillosis Centre (NAC) (Manchester, United Kingdom). The NAC is a nationally commissioned service providing long-term specialist care for patients with CPA throughout the United Kingdom. There are currently approximately 500 CPA patients on active follow-up, with approximately 130 new referrals annually. Patient sera were acquired at NAC as part of routine clinical care, and all samples were also used for measurement of *Aspergillus*-specific IgG at the time of collection. Residual sera from these routine samples were collected between September 2016 and January 2019 (>90% of the samples were collected after 1 January 2018) and stored at −80°C until use.

For each patient, CPA diagnosis was confirmed by an experienced specialist clinician. Using European Respiratory Society/European Society of Clinical Microbiology and Infectious Diseases (ERS/ESCMID) guidelines (5), diagnosis required the following combination of features: at least 3 months of relevant symptoms, characteristic radiological features, and positive “microbiological evidence.” The latter primarily consisted of a positive serological result using the Ouchterlony method to detect *Aspergillus* precipitins (10) or measurement of *Aspergillus*-specific IgG level by ImmunoCAP (positive result, >40 mgA [milligrams of antibodies]/liter IgG). The following features were also accepted as microbiological evidence: histological evidence following biopsy or resection of lung tissue, strongly positive *Aspergillus* antigen or DNA result in respiratory fluids, and microscopy of respiratory fluids showing hyphae or *Aspergillus* grown from sputum culture (5). For the CPA cases included in this study, detection of *Aspergillus* antigen was done by galactomannan EIA (Bio-Rad Laboratories, Marnes la Coquette, France), with an optical density (OD) value of >1.0 accepted as strongly positive for bronchoalveolar lavage (BAL) fluid samples and OD values of >6.5 for sputum (17). *Aspergillus* DNA in respiratory fluid (sputum) was detected using a commercially available real-time PCR diagnostic assay (ELITech, Puteaux, France), with strongly positive PCR (i.e., a transformed threshold cycle [*CT*] value of >2.0) denoting a positive result (17). In rare cases (estimated rate, 3.7% [6] to 7%), patients presented with clear clinical and radiological evidence of CPA as well as with repeatedly positive sputum *Aspergillus* culture or PCR results (ELITech, Puteaux, France), despite the absence of an antibody response (negative *Aspergillus* serology). A PCR (ELITech, Puteaux, France), culture, or galactomannan (Bio-Rad Laboratories, Marnes la Coquette, France) result was considered positive if any result in up to 5 years of patient history was positive, even if other results over that period for the same test were negative. These were also accepted as CPA cases. *Aspergillus* precipitins tests (Microgen Bioproducts, Surrey, United Kingdom) were performed for most patients at least once. Patients diagnosed with *Aspergillus* nodules were excluded (18).

Healthy control sera were obtained from the Peninsula Research Bank (Exeter, United Kingdom) and were matched by age range, ethnicity, and gender ratio to the NAC CPA patient population; donors with fungal infection and/or any condition known to predispose individuals to CPA were excluded.

### Serological analysis.

**FIG 1:**
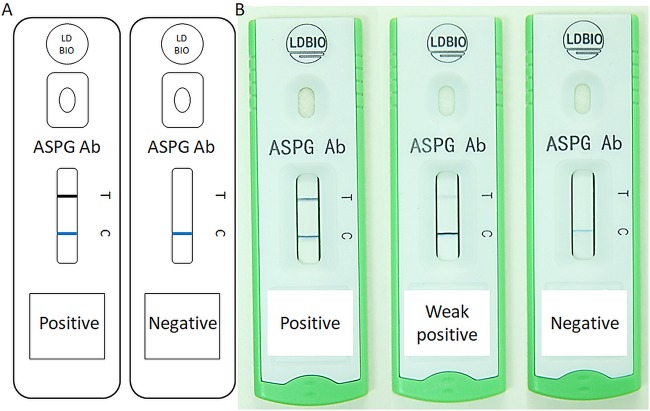
Illustrative (A) and representative (B) examples of LDBio *Aspergillus* ICT test results. ASPG Ab, *Aspergillus* antibody test.

Each sample was tested using the *Aspergillus* ICT IgG IgM lateral flow assay (LDBio, Diagnostics, Lyon, France). Test kits were shipped at ambient temperature and stored at 4°C upon receipt. Each batch (10/pack) of ICT cartridges was equilibrated to ambient laboratory temperature before use, and all tests were run according to the manufacturer’s instructions. Briefly, 15 μl of sera was dispensed onto the ICT sample application pad, followed by application of four drops of eluting solution (provided with each kit). For endpoint reads, the test was read after 20 to 30 min and results were interpreted both visually (i.e., by eye) and digitally (using a ESEQuant LR3 lateral flow reader; Qiagen, Lake Constance, Germany). Both reads were conducted by the same user, with the visual reading being conducted first to eliminate bias resulting from the digital reading. For visual reads, the test result was determined to be positive on the basis of the appearance of two lines: a blue positive-control (“C”) line and a black positive-text (“T”) line. The appearance of any black line at the “T” marker was considered to represent a positive result (Fig. 1), as recommended in the manufacturer’s guidelines. Using the LR3 lateral flow reader, a positive test result was defined by detection of peaks (any height) between 46.0 and 48.0 mm and between 53.5 and 55.5 mm for control and test lines, respectively. In rare cases, an “equivocal” result is given by the appearance of a faint, diffuse, gray line appearing where the “T” line should appear. For the purposes of this study, equivocal results, read either by eye or digitally, were excluded from the evaluation. For kinetic evaluation, the test was read digitally at automated 1-min intervals over 30 min on the LR3 reader and visually at the end of the 30-min incubation. Again, both reads were conducted by the same user. In this case, however, the digital read was conducted first and the results were shielded until after the visual read was completed. For determination of the minimum time necessary to obtain a test result, the test was read visually at 5-min intervals over 30 min and the results were scored as negative (−), weak positive (+), positive (++), or strongly positive (+++).

### Immunoblotting.

CPA sera in a randomly selected subset (*n* = 98) were also tested using another serological diagnostic assay with potential utility in resource-constrained settings, the *Aspergillus* IgG WB immunoblot assay (LDBio Diagnostics, Lyon, France). Testing was performed and results interpreted according to the manufacturer’s instructions. Briefly, 1.2 ml of sample buffer was dispensed into each channel of an incubation tray. Strips were placed into the incubation tray to rehydrate for 1 min, followed by the addition of 10 μl of serum according to the distribution plan and 90 min of incubation with agitation. A positive control, provided by the manufacturer, was included in one channel of each incubation tray. After 90 min, three washes were performed, followed by addition of 1.2ml IgG conjugate per channel and incubation for 60 min with agitation. The wash step was repeated, 1.2 ml substrate was added per channel, and the mixture was incubated for 60 min. The strips were washed twice with water and then removed to dry at room temperature for 15 min. The resulting bands on the test strips were compared to four bands on the positive-control strips, at 16, 18 to 20, 22, and 30 kDa. A positive result was defined by the presence of at least two of these bands matching the positive-control strip.

### Routine diagnostics.

*Aspergillus*-specific IgG levels were measured on all CPA patient samples as part of routine clinical care. Testing was carried out by the Manchester University NHS Foundation Trust, Department of Immunology, using an automated ImmunoCAP Phadia 1000 system (Thermo Scientific, Waltham, MA). Where a sample produced a result of >200 mgA/liter, a 1:10 dilution was performed and the sample was retested.

### Statistical analyses.

McNemar’s test was used with Yates correction (1.0) for pairwise comparisons of levels of sensitivity between LDBio *Aspergillus* ICT and ImmunoCAP tests. Pearson’s chi-square statistic was used to compare levels of sensitivity between independent sample groups. For the ICT test, we calculated Youden’s J statistic (sensitivity + specificity – 1) and the diagnostic odds ratio (DOR) (19). Binomial confidence interval (CI) (95%) data were calculated for sensitivity, specificity, and DOR (20). To compare ICT results with ImmunoCAP or WB results, we determined global concordance for all samples tested and estimated the strength of agreement using Cohen’s kappa coefficient with the following interpretations: poor (values of 0 to 0.20), fair (0.21 to 0.40), moderate (0.41 to 0.60), good (0.61 to 0.80), and very good (0.81 to 1). Spearman’s rank correlation coefficient (ρ) was used for analyses of correlation between IgG titer and ICT results. For all results, a two-tailed *P* value of <0.05 was considered statistically significant.

## RESULTS

### Patients and sera.

Patient characteristics for 154 CPA patients and 150 healthy controls are shown in Table 1. Samples for the CPA case group were collected at the NAC (Manchester, United Kingdom), and samples for the healthy control group were selected from the Peninsula Research Bank (PRB), which included samples from volunteers at the Royal Devon & Exeter NHS Foundation Trust and in community settings in and around Exeter, United Kingdom. Within the CPA patient group, two subgroups were defined according to ImmunoCAP serology results as follows: (i) a “high positive” subgroup (*Aspergillus* IgG level of >500 mg of antigen-specific antibodies [mgA]/liter) and (ii) a “seronegative” subgroup (consistent and repeated *Aspergillus* IgG level of ≤40 mgA/liter). Additionally, as part of routine testing, 145 of the 154 CPA patients in this study had microbiological culture performed on respiratory samples (sputum), usually multiple specimens. Aspergillus fumigatus was the main pathogen isolated in 84 cases (58%), other *Aspergillus* species were the main pathogens in 8 cases (6%), and no growth was observed in 53 cases (36%). Among the 84 cases positive for A. fumigatus, the samples from 29 patients also grew other *Aspergillus* species in culture. Of the 154 patients in our CPA case group, 143 had *Aspergillus* PCR performed as part of routine practice, with 74.1% sensitivity at any time point.

**TABLE 1:** Patient and control characteristics

Characteristic	Value(s)	
	CPA patients (*n* = 154)	Healthy controls (*n* = 150)
No. (%) of females	66 (42)	60 (40)
Mean age (yrs)	64	52
Age range (yrs)	32–87	36–64
No. (%) with ImmunoCAP *Aspergillus*-specific IgG result:		
>40 mgA/liter (positive result)	124 (81)	
≤40 mgA/liter (negative result)^*a*^	30 (19)	
>500 mgA/liter (high-positive result)	11 (7)	
≤40 mgA/liter (seronegative result)^*b*^	10 (7)	
No. (%) with Aspergillus fumigatus growth in sputum culture	84 (55)	
A. fumigatus only	55 (36)	
A. fumigatus + other *Aspergillus* spp.	29 (19)	
No. (%) with other *Aspergillus* (only) growth in sputum culture (A. niger [4], A. versicolor [1], A. montevidensis [1], A. insuetus [1], or A. terreus [1])	8 (5)	
No. (%) with COPD^*c*^	52 (34)	
No. (%) with prior tuberculosis	23 (15)	
No. (%) with ABPA^*d*^	19 (12)	
No. (%) with bronchiectasis	31 (20)	
No. (%) with nontuberculous mycobacterial infection	13 (8)	
No. (%) with diabetes	17 (11)	
No. (%) with sarcoidosis	16 (10)	

a Negative result for single serum sample tested.

b Negative results obtained consistently throughout patient history.

c COPD, chronic obstructive pulmonary disease.

d ABPA, allergic bronchopulmonary aspergillosis.

### ICT results.

**FIG 2:**
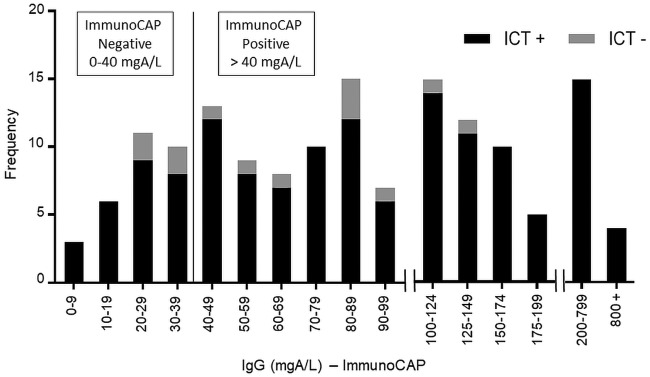
Frequency distribution of ImmunoCAP *Aspergillus*-specific IgG titers in 154 CPA patient sera tested with LDBio *Aspergillus* ICT.

The ImmunoCAP *Aspergillus* IgG distribution and the results of the ICT test are shown in Fig. 2. Of the 154 serum samples tested in the CPA patient group, 141 tested positive by ICT with 91.6% sensitivity (95% CI, 86.0% to 95.4%). In the healthy control group, 145 of the 150 sera tested negative by ICT with 98.0% specificity (95% CI, 94.2% to 99.6%). Two samples in this group yielded an equivocal result by ICT and were excluded from analysis. The routine serological test used, ImmunoCAP, was positive for 124 of the 154 CPA serum samples tested with 80.5% sensitivity (95% CI, 73.4% to 86.5%) using the current United Kingdom-approved cutoff value of 40 mgA/liter (21). A comparison of the two tests showed that the ICT test demonstrated higher sensitivity than the ImmunoCAP in detecting *Aspergillus* antibody (McNemar’s *P = *0.007). Results were in agreement for 119 of the 154 samples tested (77.3%) with a Cohen’s kappa coefficient of 0.077 (95% CI, −0.087 to 0.241), indicating poor agreement between the ICT and ImmunoCAP results. Using other suggested cutoff values of 20 mgA/liter (6) and 50 mgA/liter (22), the ImmunoCAP sensitivities for this data set were calculated as 93.5% (95% CI, 88.4% to 96.8%) and 71.4% (95% CI, 63.6% to 78.4%), respectively, with the ICT test having significantly better performance than ImmunoCAP at a cutoff value of 50 mgA/liter (McNemar’s *P < *0.001) but with no difference seen at 20 mgA/liter. Of the 154 patients in our CPA case group, 108 had precipitins testing (for *Aspergillus* antibody) performed as part of routine diagnostics, with 57.4% sensitivity.

For the high-positive CPA cases, 11 of the 11 serum samples tested positive by ICT with 100% sensitivity (95% CI, 71.5% to 100%), indicating a low likelihood of prozone effect at higher levels of circulating *Aspergillus* IgG. In the seronegative CPA patient subset, 9 of the 10 serum samples tested positive by ICT with 90.0% sensitivity (95% CI, 55.5% to 99.8%). Youden’s J statistic calculated for the ICT test results indicated a good balance between sensitivity and specificity, and the test had a high DOR of 524 (95% CI, 146 to 1,879) (Table 2). All tests were read both visually (i.e., by eye) and digitally (by the use of a Qiagen ESEQuant LR3 lateral flow reader), and the results from these two methods had 100% agreement. For 5 of the 154 CPA sera tested (3.3%), only a very faint band was present, making the result difficult to detect by eye. When the appearance of a band was in question, the test was read vertically above the reading area and near a window or in direct light, as recommended by the manufacturer for bands of very weak intensity. In these cases, results were also confirmed visually by a second reader and digitally using the test-specific optimized parameters on the LR3 reader, and both results were confirmed with 100% agreement.

**TABLE 2:** Summary of results for LDBio *Aspergillus* ICT IgG-IgM test and routine serology^*a*^

Test	% sensitivity (95% CI)			% specificity (95% CI) (healthy controls [*n* = 148])^*c*^	Youden's index (all sera [*n* = 302])^*d*^	DOR (95% CI) (all sera [*n* = 302])
	All CPA (*n* = 154)	High positive (*n* = 11)^*b*^	Seronegative (*n* = 10)^*b*^			
LDBio ICT	91.6 (86.0, 95.4)	100 (71.5, 100)	90.0 (55.5, 99.8)	98.0 (94.2, 99.6)	0.896	524 (146, 1,879)
ImmunoCAP	80.5 (73.4, 86.5)	100 (71.5, 100)	0 (0, 30.9)	NA^*e*^	NA	NA
McNemar's (*P* value)	0.007			NA		

a Results are reported against the cutoff of 40 mgA/liter recommended for use in United Kingdom laboratories at the time of publication.

b ImmunoCAP results were used to define the “high positive” and “seronegative” groups.

c Equivocal results (*n* = 2) were excluded from analysis.

d Youden's index = sensitivity + specificity − 1.

e NA, not applicable.

In relation to *Aspergillus* species detected in sputum from CPA patients (Table 3), the ICT test performed best for A. fumigatus cases (with 96.4% sensitivity) but also detected antibodies in most cases where only non-fumigatus *Aspergillus* species had been isolated (with 87.5% sensitivity), with no significant differences in sensitivity seen between these groups (Pearson’s chi-square statistic, 1.4002; *P = *0.24). Overall, the sensitivity seen with samples from patients with at least one *Aspergillus* species isolated by culture was better than that seen with samples from patients that showed no growth in sputum culture (95.7% and 88.7%, respectively), but this did not represent a significant difference (Pearson’s chi-square statistic 2.5464, *P = *0.11). Among the samples from 9 patients for whom no sputum culture was conducted, 6 were positive by ICT (66.7% sensitivity; 95% CI, 29.9% to 92.5%).

**TABLE 3:** LDBio *Aspergillus* ICT performance in CPA cases with Aspergillus fumigatus and non-A. fumigatus species

Sputum culture result (*n* = 145)	*n*	ICT^+^ (*n*)	% sensitivity (95% CI)
All *Aspergillus* growth	92	88	95.7 (89.2, 98.8)
A. fumigatus	84	81	96.4 (89.9, 99.3)
A. fumigatus only	55	54	98.2 (90.3, 100)
A. fumigatus + other *Aspergillus* spp.	29	27	93.1 (77.2, 99.2)
Other *Aspergillus* spp.	8	7	87.5 (47.4. 99.7)
A. niger	4	3	
A. insuetus	1	1	
A. montevidensis	1	1	
A. terreus	1	1	
A. versicolor	1	1	
			
No aspergillus growth	53	47	88.7 (77.0, 95.7)

### ICT agreement with immunoblotting.

A random sampling of 98 CPA patient sera from the 154 tested by ICT was also tested by LDBio *Aspergillus* WB immunoblotting for detection of *Aspergillus*-specific IgG. Sensitivity levels were not significantly different between the two tests (McNemar’s *P = *0.504). Results were in agreement for 89 of the 98 samples tested (90.8%), with Cohen’s kappa coefficient indicating moderate agreement between the ICT and immunoblotting results (Table 4).

**TABLE 4:** Summary of ICT and immunoblot results in a randomly selected subset of 98 sera from patients with CPA

Test	No. of sera with positive result (*n* = 98)	% sensitivity (95% CI)	% agreement	Cohen's kappa (95% CI)
ICT	88	89.8 (82.0, 95.0)	90.80	0.558 (0.301, 0.814)
Immunoblot	85	86.7 (78.4, 92.7)		

### ICT result correlation with *Aspergillus* IgG titer.

**FIG 3:**
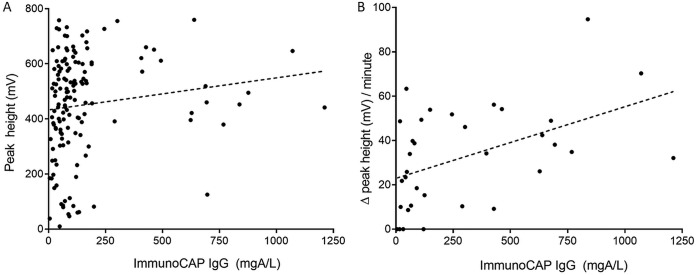
Correlation between ImmunoCAP *Aspergillus*-specific IgG titer and LDBio *Aspergillus* ICT test bands read on a Qiagen ESEQuant LR3 lateral flow reader. There was a weak correlation between IgG titer and test band peak height (ρ = 0.2821, *P = *0.003) (A), and there was a moderate correlation between IgG titer and the rate of test band development (initial rate *t* = 0 to 5 min) (ρ = 0.4927, *P* = 0.003) (B).

**FIG 4:**
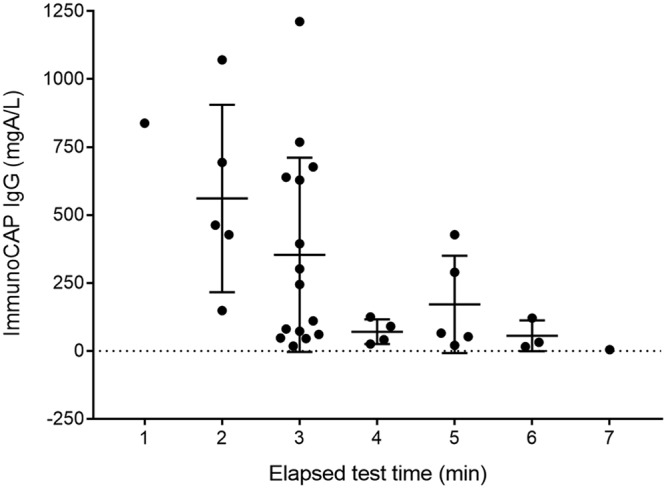
Distribution of ImmunoCAP *Aspergillus*-specific IgG results in relation to elapsed time to the first appearance of a test line on LDBio *Aspergillus* ICT.

Using a Qiagen LR3 reader, the peak height of the ICT test line was recorded at the time of the reading of the test results, which was between 20 and 30 min as recommended by the manufacturer, for all samples from the members of the healthy control and CPA case groups. Using positive results from the CPA case group (*n* = 141), a weak but significant correlation was found between endpoint peak height and ImmunoCAP IgG titer (Spearman’s rank correlation coefficient ρ = 0.2821, *P = *0.003) (Fig. 3A). We also determined the rate of ICT test line development for a subset of positive results from the CPA case group (*n* = 38), using peak height readings taken at automated 1-min intervals for 30 min on the LR3 reader. The initial rate (change in peak height per minute) over the first 5 min of test line development was calculated, and a moderate correlation was found between the initial rate of test line development and ImmunoCAP IgG titer (Spearman’s rank correlation coefficient ρ = 0.4927, *P = *0.003) (Fig. 3B). Initial rates were also evaluated for the first 7 and 10 min of the test, and the evaluations yielded similar results (Spearman’s rank correlation coefficient ρ = 0.4553 and *P = *0.007 [7 min]; ρ = 0.4225 and *P = *0.013 [10 min]). Finally, we compared the elapsed ICT test time to the time of the first appearance of the test band with ImmunoCAP IgG titer for the same subset (Fig. 4). Although there was a trend for test lines to appear earlier for high-positive samples (IgG, >500 mgA/liter), the results showed no significant difference between time points.

### Effect of elapsed time on ICT accuracy.

**FIG 5:**
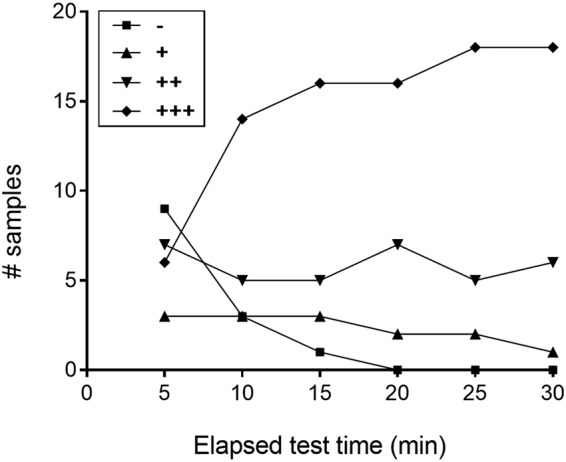
Number of positive serum results showing negative (−), weak positive (+), positive (++), or strongly positive (+++) results at each 5-min read interval for the LDBio *Aspergillus* ICT test.

A total of 28 sera from the CPA case group were randomly selected to run on the ICT test, the test was read visually at 5-min intervals over 30 min, and the results were scored as negative (−), weak positive (+), positive (++), or strongly positive (+++). The ICT reached maximum sensitivity at 20 min (Table 5). Examining the development of test bands in the positive result set (*n* = 25) during the 30-min incubation, all positive tests exhibited visible test lines by 20 min that were stable to the 30-min endpoint (Fig. 5).

**TABLE 5:** Sensitivity of ICT versus elapsed ICT run time

Elapsed time (min)	% sensitivity (95% CI)
5	57.1 (37.2, 75.5)
10	78.6 (59.1, 91.7)
15	85.7 (67.3, 96.0)
20	89.3 (71.8, 97.7)
25	89.3 (71.8, 97.7)
30	89.3 (71.8, 97.7)

## DISCUSSION

While CPA is regarded as a rare disease in high-income countries, its burden in low-income and middle-income countries with high incidences of pulmonary tuberculosis (TB) is considerable. An estimate for India put the 5-year prevalence at 290,147 cases, or 24 per 100,000 (23), and for Pakistan at 72,438 cases, or 39 per 100,000 population (24). Put another way, a prospective study in Uganda in both human immunodeficiency virus (HIV)-infected and uninfected patients found an annual rate of CPA development of 6.5% in those with a residual cavity at the end of TB treatment, typically found in 22% to 35% of cases (7). It is likely that substantial numbers of patients are incorrectly diagnosed as having pulmonary TB rather than CPA, as was found in 19% of the members of a group of HIV-negative, GeneXpert-negative, and smear-negative patients in Nigeria (25). About 45% of the global population of patients with pulmonary TB, representing around 2.5 million cases, are currently not confirmed microbiologically (26). Availability of a simple, easy-to-use assay for *Aspergillus*-specific antibody will be of great value in establishing an accurate diagnosis of subacute and chronic pulmonary infection.

The recently introduced LDBio *Aspergillus* ICT test requires minimal time, resources, and equipment, and its use would be highly compatible with settings where CPA diagnostics are a critical need. Our evaluation has shown very good sensitivity (91.6%) and specificity (98.0%) for diagnosis of CPA in United Kingdom patients, with the ICT test significantly outperforming our workhorse assay—the ImmunoCAP test. In subsets of the CPA cohort, the LDBio ICT test performed similarly to the LDBio *Aspergillus* WB immunoblot test, with moderate agreement, but had increased sensitivity over PCR and precipitins testing. There was no prozone effect at high *Aspergillus* IgG titers, and the ICT test was able to accurately identify clinically confirmed cases of CPA where ImmunoCAP repeatedly failed to do so. One possible explanation for the differences in performance between the ICT test and the ImmunoCAP test, both overall and for the seronegative subset, might be differences in the mixtures of antigen used to capture *Aspergillus* antibody. Additionally, the ImmunoCAP test is IgG specific, whereas the LDBio *Aspergillus* ICT test is claimed to detect both IgG and IgM in patient sera. While elevated levels of IgG are found in nearly all cases of CPA (8, 10, 27), *Aspergillus*-specific IgM has also been detected in up to 50% of CPA cases (28–32). Though raised levels of specific IgM are often associated with acute infection, *Aspergillus*-specific IgM in cases of CPA may respond dynamically to the various antigens that *Aspergillus* produces at different stages of its growth cycle during chronic infection (33). As such, it is probable that an assay using a mixture of antigens could remain positive for IgM over an extended period of infection (11). One limitation of *Aspergillus*-specific IgM testing is poor specificity (28, 30, 34, 35); however, the ability of the LDBio *Aspergillus* ICT test to detect both IgG and IgM may overcome this constraint to detect variations in serological responses to CPA. This study did not evaluate the individual contributions of IgG and IgM from patient sera to the ICT results acquired, and there have been no published studies on the antibody class-specific performance of the *Aspergillus* ICT test, warranting further investigation.

The majority of CPA cases in this study were culture positive for A. fumigatus. A limited number of non-A. fumigatus CPA cases were evaluated, and we found no significant differences in ICT performance between A. fumigatus culture-positive cases and non-A. fumigatus cases. This represents a particularly important consideration for selecting a diagnostic assay for use in resource-constrained settings, where CPA caused by non-A. fumigatus *Aspergillus* species—particularly A. niger (25, 36) and A. flavus (37)—is more prevalent. Other commonly used serological assays are primarily specific for A. fumigatus and do not exhibit *Aspergillus* species cross-reactivity (38, 39) or have not been evaluated for their utility in diagnosing CPA caused by non-A. fumigatus strains (22, 40). Further assessment of the LDBio ICT test for *Aspergillus* species cross-reactivity is necessary, but these preliminary results indicate that it may be more useful than current tests to diagnose CPA in countries where aspergillosis species epidemiology varies.

In addition to their utility in diagnosing CPA, serum levels of *Aspergillus*-specific IgG have been successfully used to monitor CPA responses to clinical treatment or surgical resection (8, 41–45). We sought to determine if the LDBio *Aspergillus* ICT test could be useful not only as screening test for CPA but also for quantification or semiquantification of IgG levels. Although weak to moderate correlations were found between ImmunoCAP IgG titer and ICT test line intensity (peak height) or rate of test line development, neither correlation was sufficient for reliable quantification of *Aspergillus*-specific IgG using test line measurements. Finally, using a selection of CPA samples, we determined that peak sensitivity for the ICT test occurs at 20 min after sample addition. The appearance of a test line and a control line before this time point can be recorded as a positive result; however, a negative result cannot be accurately determined until at least 20 min of incubation has been performed due to the slower appearance of weakly positive lines. In these cases, it is recommended to wait the full 30 min, as a small percentage of our positive-testing CPA sera (5/154, 3.3%) analyzed using the ICT test resulted in only a faint appearance of a test line, and such lines may be challenging to detect and slow to develop.

A possible limitation of this study is the use of healthy blood donors as controls. While adequate to provide a baseline for test performance, they do not accurately represent the population and setting in which the test is most likely to be used, where underlying respiratory disease and/or other sources of pulmonary infection are common (1, 2, 46). Human immunodeficiency virus (HIV) infection is also frequently comorbid with TB (47), the most common underlying disease in CPA in low-income and middle-income countries, but the effect of HIV on IgG response to *Aspergillus* is uncertain (7, 25), although most cases appear to be detected. In the United Kingdom and probably other high-income countries, chronic obstructive pulmonary disease (COPD) cases outnumber TB cases as the predominant underlying disorders (44, 46, 48), and since COPD is common but CPA unusual, highly discriminatory tests are required. Respiratory culture is insensitive (49), as is serum galactomannan and beta 1,3-d-glucan detection (5, 50–52). *Aspergillus* antibody detection is critical to establishing a CPA diagnosis (5). The current workhorse test, ImmunoCAP, has the advantages of being semiautomated and quantitative. We have previously found it to have sensitivity and specificity of 96% and 98%, respectively, using a much lower cutoff value of 20 mgA/liter with Ugandan controls (4, 6) and to be 84% sensitive and 98% specific using a cutoff value of 50 mgA/liter with a different set of (European) controls (22). In Japan, the ImmunoCAP results showed 98% sensitivity and 84% specificity in a large cohort of patients with respiratory disease but, critically, the results were determined using a positive ImmunoCAP test result as a component of the diagnosis of CPA (40). In India, 137 CPA patients and healthy controls were studied, and the optimum cutoff of the ImmunoCAP assay was 27 mgA/liter, with a sensitivity and specificity of 91 to 96% and 100%, respectively (53). Those studies demonstrated that both the cutoff value and the control population significantly affected diagnostic performance. Additional evaluation of the LDBio *Aspergillosis* ICT test using diseased controls would be beneficial to truly establish its specificity and utility for diagnosing CPA. Further studies are also necessary to determine batch-to-batch variation and reproducibility of ICT results, and, based on our observation that test and control lines persist beyond the recommended reading window (20 to 30 min), the validity of data determined beyond 30 min should be assessed as well.

The strength of our study was that the microbiological element used for CPA diagnosis was based on multiple tests and not reliant only on serology. Using these clinically and microbiologically defined CPA cases, we found the LDBio *Aspergillus* ICT test to have good sensitivity and specificity. The test is easy to perform, and we found visual interpretation to be as reliable as digital detection. Furthermore, the test has been shown to operate reliably under conditions of high temperature and humidity (16) and requires minimal laboratory equipment and no power source, important features for implementing diagnostics in resource-constrained settings. While not quantitative and therefore not suitable for monitoring CPA treatment, overall, the LDBio *Aspergillus* ICT test exhibits excellent performance as a screening tool in the CPA diagnostic pathway.
